# Dysfunction of the ABCA1 and ABCG1 Transporters and Their Impact on HDL Metabolism

**DOI:** 10.3390/antiox14111362

**Published:** 2025-11-14

**Authors:** Kevin David Laguna-Maldonado, Daniel Uribe-Ramírez, Melissa Vázquez-Carrada, Deyamira Matuz-Mares, María Magdalena Vilchis-Landeros

**Affiliations:** 1 Departamento de Bioquímica, Facultad de Medicina, Universidad Nacional Autónoma de Mexico, Ciudad de Mexico C.P. 04510, Mexico; kevinlag@facmed.unam.mx (K.D.L.-M.); daniel.uriberam@gmail.com (D.U.-R.); deya@bq.unam.mx (D.M.-M.); 2 Institute of Microbiology, Cluster of Excellence on Plant Sciences, Heinrich Heine University Düsseldorf, 40225 Düsseldorf, Germany; m.vazquez-carrada@hhu.de

**Keywords:** ABC transporters, ABCA1, ABCG1, HDL, atherosclerosis, oxidative stress

## Abstract

High-density lipoprotein (HDL) metabolism depends on several key factors, including ATP-binding cassette (ABC) transporters such as ABCA1 and ABCG1. These transporters are essential for maintaining cholesterol homeostasis by mediating the efflux of cellular lipids and promoting HDL formation and maturation. Dysfunction in these pathways compromises HDL biogenesis, leading to lipid accumulation in macrophages and peripheral cells. Together with oxidized low-density lipoproteins (LDLs), these alterations promote foam cell formation, atherosclerotic plaque development, and the progression of cardiovascular and metabolic diseases. Oxidative stress plays a central role in disturbing lipid balance and impairing ABC transporter activity. Unlike previous reviews that have mainly summarized mechanisms of oxidative regulation, this work integrates recent molecular findings to propose a unifying framework in which oxidative stress sequentially disrupts ABCA1 and ABCG1 function, thereby altering HDL metabolism. Moreover, it highlights emerging pharmacological strategies aimed at restoring cholesterol homeostasis and mitigating oxidative damage, contributing to the prevention of cardiovascular and metabolic disorders.

## 1. Introduction

Lipids constitute one of the principal structural and functional components of living organisms. Beyond being a fundamental source of metabolic energy, they play essential roles in cell membrane architecture, molecular signaling, and the regulation of energy metabolism [1,2,3]. Under physiological conditions, the balance between lipid synthesis, transport, and degradation is essential to maintaining metabolic homeostasis and preventing the onset of chronic diseases. However, this delicate equilibrium can be disrupted by several factors, among which oxidative stress stands out, as it compromises both the structural integrity of lipids and their metabolic regulation [4,5,6,7].

Lipid transport is mediated by lipoproteins that enable hydrophobic molecules, such as triacylglycerols and cholesterol, to circulate in the aqueous environment of plasma [8,9]. These lipoproteins, chylomicrons (CMs), very-low-density lipoproteins (VLDLs), low-density lipoproteins (LDLs), and high-density lipoproteins (HDLs), ensure the proper delivery of lipids to peripheral tissues and the clearance of excess cholesterol to the liver through the process known as reverse cholesterol transport (RCT) [10,11,12,13,14,15,16,17]. Apolipoproteins, including ApoA-I, ApoB-100, and ApoE, are essential structural and functional components of lipoproteins [18,19]. They facilitate lipid binding, mediate recognition by cellular receptors, and modulate the activity of enzymes involved in lipoprotein metabolism, thereby ensuring efficient lipid transport and metabolic regulation [12,20,21]. Among these lipoproteins, HDL particles play a particularly protective role by facilitating cholesterol efflux from peripheral cells, thereby preventing foam cell formation and the progression of atherosclerosis [22,23,24,25,26].

Lipoprotein metabolism involves a complex network of apolipoproteins, enzymes, and transporters that regulate lipid uptake, efflux, and transformation at the cellular level [8,13]. There, the ATP-binding cassette (ABC) transporters, particularly ABCA1 and ABCG1, are crucial for mediating cholesterol efflux and phospholipid transfer to HDL particles, thereby enabling their formation and maturation [27,28]. Dysfunction of these transporters, whether caused by genetic variations or environmental factors, impairs RCT and promotes intracellular cholesterol accumulation, especially in macrophages, leading to foam cell formation and the development of cardiovascular diseases such as atherosclerosis [28,29], in which oxidative stress plays a central role. Oxidative stress is defined as an imbalance between the generation of reactive oxygen species (ROS) and the organism’s antioxidant defense systems [4,5]. Due to cellular metabolism, ROS are generated from different compartments such as mitochondria, peroxisomes, endoplasmic reticulum, cytosolic enzymatic systems, and exogenous factors [30].

Excessive ROS not only induce lipid peroxidation and structural cellular damage but also modify the ABCA1 and ABCG1 transporters, reducing their interaction with apolipoproteins and consequently their efficiency in mediating cholesterol efflux [31,32]. In this way, oxidative stress links lipid metabolism with inflammation and vascular dysfunction, representing a key pathogenic axis in the progression of metabolic and cardiovascular diseases [33,34,35,36,37,38].

A comprehensive understanding of the interplay between lipid metabolism, cholesterol transport, and the oxidative modulation of ABCA1 and ABCG1 transporters thus provides an essential framework for elucidating novel therapeutic strategies aimed at preventing and treating atherosclerosis and related metabolic disorders [28,29].

This article aims to explain the relationship between oxidative stress and lipid homeostasis, showing how ROS damage to the ABCA1 and ABCG1 transporters disrupt RCT, thereby contributing to the development and progression of atherosclerosis and other metabolic disorders. Furthermore, it discusses current and emerging therapeutic strategies focused on restoring lipid balance and preventing disease progression.

## 2. ABC Transporters

ABC transporters constitute one of the largest and most diverse protein superfamilies in living beings: they are essential for their role as primary active transporters [39]. They use the energy generated by ATP hydrolysis to move molecules across biological membranes, thus serving for the translocation of various substrates, including vitamins, steroids, lipids, ions, peptides, proteins, polysaccharides, and xenobiotics [40,41,42]. Currently, 48 ABC transporters have been identified in humans, which have been classified into 7 subfamilies: A, B, C, D, E, F, and G [40]. The importance of this protein family lies in its functional variety and its implications in various pathologies, including cystic fibrosis, cancer, adrenoleukodystrophy, retinal degeneration, and cholesterol transport defects [42,43,44].

In general, human ABC transporters are located at the plasma membrane and export substrates from the cytoplasm. However, some are in peroxisomes, lysosomes, photoreceptor discs, and mitochondria [45,46].

According to their structure, ABC transporters can be organized in two ways: as a complete transporter formed by a monomer, which is the case of ABCA1, or as a semi-transporter formed by dimers (homodimers or heterodimers), which is the case of ABCG1 [40,47,48]. These transporters possess ATP-binding and hydrolysis sites, known as nucleotide-binding domains (NBDs), and are highly conserved [49]. NBDs contain the consensus sequences Walker A (phosphate-binding) and Walker B (ATP-binding and hydrolysis) [47,50]. They also contain transmembrane domains (TMDs), each comprising several hydrophobic α-helices, which are involved in substrate recognition and translocation across the lipid membrane [51].

ABC transporters participate in numerous cellular processes such as the maintenance of osmotic homeostasis (ABCC7) [52], antigen processing (ABCB2/3) [39], cell proliferation (ABCC1, ABCG2, ABCB6) [44,53,54], the immune response (ABCB2/3) [55] and the efflux of cholesterol and lipids (ABCA1 and ABCG1) [56,57].

**Figure 1:**
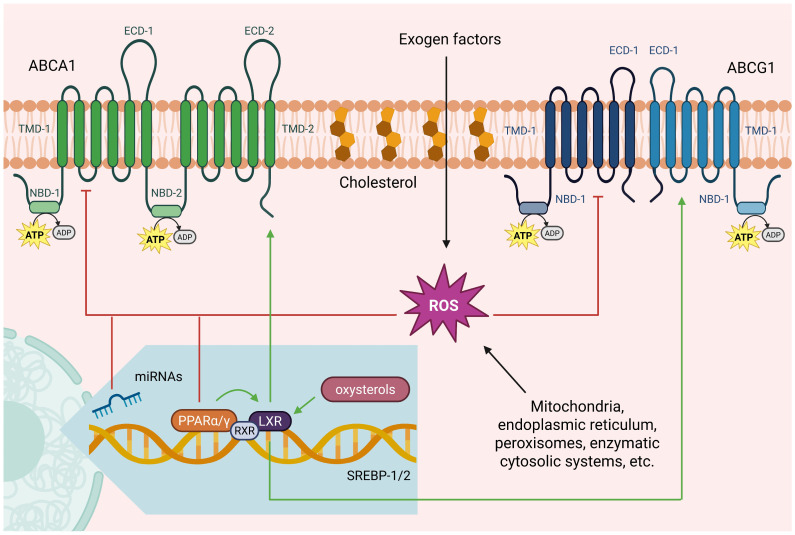
Structure and regulation of ABCA1 and ABCG1. ABCA1 is a complete transporter composed of a single polypeptide containing two ATP-binding sites (NBDs), two transmembrane domains (TMDs) each with six α-helices, and two extracellular domains (ECDs) that are essential for ApoA-I binding. In contrast, ABCG1 is a semi-transporter that functions as a dimer. Each monomer consists of one NBD, one TMD, and one ECD. These transporters are finely regulated. Their function is inhibited by ROS produced through various intracellular and extracellular sources. While their expression is induced via nuclear hormone receptors such as the liver X receptor (LXR), the retinoid X receptor (RXR) and the peroxisome proliferator-activated receptors alpha and gamma (PPARα and PPARγ), and it is inhibited by microRNAs located in the intronic regions of genes encoding sterol regulatory element binding factors 1 and 2 (SREBF1 and SREBF2). Created in BioRender. Matuz Mares, D. (2025) https://BioRender.com/5kwwntp.

ABCA1 and ABCG1 act primarily as translocators of sterols and lipids from plasma membranes to lipoproteins, particularly HDLs [57]. They are ubiquitously expressed, although there is higher expression in some cells or tissues (Table 1) [40,58]. The function of these two transporters is vital, because they prevent the accumulation of lipids in macrophages, hepatocytes, enterocytes, endothelial cells and vascular smooth muscle cells [56,57,58,59,60,61,62,63]. ABCA1 is composed of 2261 amino acid residues that form six domains: two TMDs consisting of six transmembrane helices each; two NBDs in the cytoplasm, which serve to couple ATP hydrolysis to translocase activity, and two large extracellular domains (ECDs) that are involved in protein–protein interactions, essential for ApoA-I binding and regulatory functions (Figure 1) [59,64].

On the other hand, ABCG1 can be composed of 666 or 678 amino acids, depending on alternative splicing, leading to the formation of two isoforms: ABCG1 (−12) and ABCG1(+12), respectively [47]. It has only one NBD and a TMD with six transmembrane helices, which dimerize to form a functionally active transporter (Figure 1) [48].

Mutations in the *abca1* gene have been reported to cause the autosomal recessive genetic disorder called Tangier disease [65], in which patients show very low serum HDL levels, increasing the risk of atherosclerosis (Table 1). No pathogenic alterations in the *abcg1* gene have been identified in humans. Although in murine models, in which the *abcg1* gene has been eliminated and they are fed with a diet high in fat and cholesterol, show accumulation of lipids in hepatocytes and macrophages [66]. In humans, alterations in the function of both transporters are related to the development of various diseases, as mentioned in Table 1.

**Table 1:** Characteristics of ABCA1 and ABCG1 transporters.

Transporter	ABCA1	ABCG1
Type of transporter	Full transporter [40,48]	Half transporter
Molecular Mass	254 kDa [40]	75.69 kDa [40]
Tissue expressed	Ubiquitous, highly expressed in hepatocytes, macrophages, and smooth muscle cells [67,68]	High expression levels in macrophages, lymphocytes, epithelial cells, endothelial cells, vascular smooth muscle cells, liver, and intestine, brain and placenta [60,62,69,70,71]
Cell localization	Plasma membrane, endosome, peroxisomes, mitochondria, endoplasmic reticulum, or lamellar bodies [40]	Plasma membrane, endosomes, peroxisomes, mitochondria, endoplasmic reticulum, Golgi apparatus, lamellar bodies and endocytic vesicles [40,72]
Function	HDL biogenesis, cholesterol efflux, insulin secretion, microvesicle formation in platelets, glucose uptake in skeletal muscle, apolipoprotein ApoE secretion, and lipidation in astrocytes [67]	Transport of cholesterol, phosphatidylcholine, sphingomyelin, oxysterol and participates in RCT [47,63,73]
Human Genetic disease	Tangier disease [65]	There are no reports [40]
Associated diseases	Dyslipidemia, atherosclerosis, inflammation, coronary heart disease, type 2 diabetes, thrombosis, neurological disorders, age-related macular degeneration, glaucoma, viral infections, and cancer progression [32,67,74,75]	Atherosclerosis, inflammation, Alzheimer’s disease (AD), type 2 diabetes, cancer, immune disorders, obesity, and age-related macular degeneration [47,60,63,71]

The expression of these transporters is an activation cascade involving different elements. It begins with the activation of some nuclear hormone receptors, such as liver X receptor (LXR) and retinoid X receptor (RXR) [25,48,76], which in turn are activated by fatty acids and oxysterols. (Figure 1). Fatty acids positively stimulate peroxisome proliferator-activated receptors alpha and gamma (PPARα and PPARγ) [77,78], while oxysterols act directly on nuclear receptors LXR and RXR [79,80]. These same transporters are negatively regulated through microRNAs (miRNAs/miR) such as miR-33a and miR-33b [81], which are located in intronic regions of sterol regulatory element binding factor 1 and 2 (SREBF1 and SREBF2) genes, which control cholesterol synthesis [82]. Finally, at the post-translational level, both transporters are degraded by the calpain and proteasome pathways in macrophages [48,83,84].

## 3. HDL Metabolism

**Figure 2:**
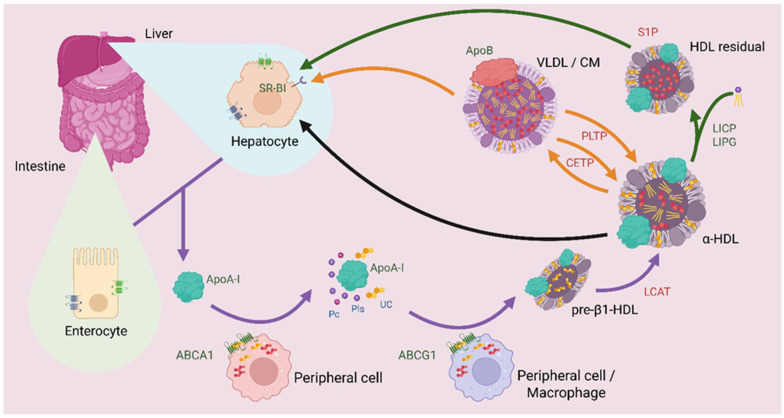
HDL metabolism. Purple arrows illustrate the process of HDL formation and maturation. It begins with the release of ApoA-I from hepatocytes or enterocytes. ApoA-I circulates through the bloodstream to peripheral cells expressing the ABCA1 transporter, where it acquires phosphatidylcholine (PC), unesterified cholesterol (UC), and phospholipids (PLs). Subsequently, pre-β1-HDL interacts with macrophages or other peripheral tissues expressing ABCG1, where it again acquires PC, UC, and PLs to form a larger particle. Then, LCAT esterifies cholesterol, transforming pre-β1-HDL into α-HDL, the mature form of HDL. The black arrow represents the reverse cholesterol transport (RCT) pathway, in which α-HDL returns cholesterol to hepatocytes through recognition by the scavenger receptor class B type 1 (SR-B1) to allow cholesterol metabolism. The orange arrows indicate a possible fate of α-HDL, in which cholesteryl ester transfer protein (CETP) and phospholipid transfer protein (PLTP) exchange cholesteryl esters and triacylglycerols among very-low-density lipoproteins (VLDL), chylomicrons (CM), and α-HDL. This enables VLDL and CM to deliver more cholesterol to peripheral tissues, while α-HDL returns triacylglycerols to the liver. The green arrows represent another remodeling pathway of α-HDL, in which interaction with hepatic lipase (LIPC) and endothelial lipase (LIPG), which are in the bloodstream, reduces the triacylglycerol content of α-HDL, generating HDL residual enriched in sphingosine-1-phosphate (S1P) that are subsequently returned to hepatocytes for metabolism. Created in BioRender. Matuz Mares, D. (2025) https://BioRender.com/fpnzf7i.

The HDL metabolism starts with the synthesis and secretion of ApoA-I by hepatocytes and enterocytes, the main structural apolipoprotein of HDL [22]. Free ApoA-I in plasma interacts specifically with the ABCA1 transporter on the membrane of peripheral cells, particularly macrophages and endothelial cells, initiating the assembly of HDL (Figure 2) [85]. ABCA1 utilizes the energy from ATP hydrolysis to translocate phospholipids (primarily phosphatidylcholine) and unesterified cholesterol from the cell membrane to ApoA-I, thereby generating small discoidal HDL particles known as pre-β1-HDL. Instead, ABCG1 facilitates the efflux of cholesterol and phospholipids to pre-β1-HDL, forming a bigger particle [19,48]. Seemingly, ABCG1 is more efficient in transferring lipids to pre-β1-HDL, whereas ABCA1 primarily loads lipids onto lipid-free ApoA-I [86]. This indicates that, unlike ABCA1, ABCG1 does not initiate HDL biogenesis de novo, but acts at a later stage of the HDL cycle, promoting their lipid enrichment [25].

Newly formed pre-β1-HDL undergoes a maturation process through the sequential action of multiple plasma factors (Figure 2): Lecithin cholesterol acyltransferase enzyme (LCAT), activated by ApoA-I, esterifies the captured cholesterol using a fatty acid from phosphatidylcholine, generating cholesterol esters that accumulate in the hydrophobic core of the particle and transforming its morphology from discoidal to spherical (α-HDL) [87].

α-HDL circulate in plasma, where they undergo continuous remodeling via three principal pathways (Figure 2): (1) cholesteryl ester transfer protein (CETP) and phospholipid transfer protein (PLTP) mediate the exchange of cholesteryl esters for triacylglycerides and phospholipids with triacylglycerol-rich lipoproteins (VLDL and CM); (2) Hepatic lipase (LIPC) and Endothelial lipase (LIPG) selectively hydrolyze triacylglycerides and phospholipids to modify α-HDL composition; and (3) the Scavenger Receptor Class B type I (SR-BI) in hepatocytes mediates the selective uptake of cholesteryl esters without degrading the entire particle [88,89]. This last mechanism represents the final stage of RCT, where cholesterol is returned to the liver for biliary excretion or metabolic reuse [22].

Additional to its role in RCT, ABCG1 contributes to cholesterol synthesis by regulating sterol regulatory element-binding protein 2 (SREBP-2), which activates the expression of genes such as β-hydroxy β-methylglutaryl-CoA (HMG-CoA), HMG-CoA reductase (HMGR), HMG-CoA synthase (HMGS), squalene synthase (Sqs), farnesyl diphosphate synthase (Fpps), the low-density lipoprotein receptor (LDLR), and other genes involved in cholesterol synthesis and transport, including insulin-induced gene 1 protein (INSIG1) and StAR-related lipid transfer protein 4 (StarD4) [90].

## 4. Alterations in HDL Metabolism

The transporters involved in cholesterol efflux systems are particularly vulnerable to the prooxidant microenvironment characteristic of conditions such as atherosclerosis, diabetes, and metabolic syndrome. For example, a study in vivo in murine blood cells has demonstrated that NOX enzymes (NOX 2/4), are a major contributor to ROS production, leading to the downregulation of transporters such as ABCA1 and ABCG1, and induce the accumulation of oxidized LDL, especially in areas susceptible to atherosclerosis [91,92,93]. Additionally, ABCG1 expression was downregulated in human umbilical cord cells exposed to a prooxidant microenvironment, leading to an increase in the activity of NADPH oxidase (particularly NOX4) and reduced the levels of Nuclear factor erythroid 2 (Nrf2), a key regulator of antioxidant defense genes [63].

**Figure 3:**
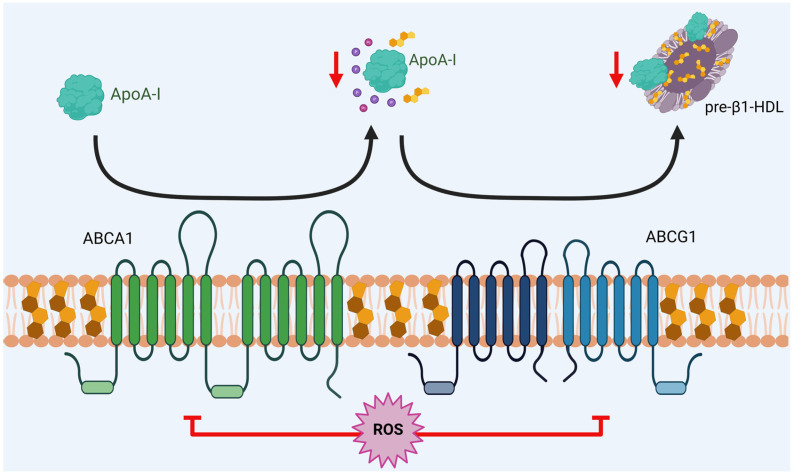
Dysfunction of HDL metabolism. The generation of ROS reduces the function of ABCA1 and ABCG1 transporters, leading to a decrease in ApoA-I lipidation and producing a similar effect on the formation of pre-β1-HDL and α-HDL (mature HDL) particles. This reduction leads to impaired reverse cholesterol transport (RCT). Created in BioRender. Matuz Mares, D. (2025) https://BioRender.com/efnkqlg.

In addition, the decreased formation of new HDL particles and alterations of existing HDL reduce the efficiency of RCT by decreasing ABCA1 activity, compromising the initial formation of pre-β1-HDL particles, which serve as the primary acceptors of cholesterol from peripheral tissues (Figure 3). When this rate limiting step is impaired, intracellular accumulation of unesterified cholesterol occurs, particularly in macrophages within the arterial intima, promoting foam cell formation [94]. Also, when α-HDL are in an oxidative environment undergo structural modifications that render them dysfunctional: oxidation of methionine residues in ApoA-I (particularly Met^148^ and Met^158^). This compromises its ability to activate the enzyme LCAT, which is essential for cholesterol esterification and HDL maturation [95,96]. Furthermore, oxidized HDL exhibits an increased affinity for CETP, promoting the transfer of oxidized lipids to LDL. This phenomenon creates a vicious cycle, as LDL modified through this process becomes more susceptible to further oxidation and more atherogenic [91,97].

Also, when the LDL are oxidized, they are not efficiently recognized by the conventional LDLR, but they are preferentially taken up by Scavenger Receptor Class A type I (SR-A1) and Platelet glycoprotein 4 (CD36) on macrophages, a process that is not subject to negative feedback regulation by intracellular cholesterol [19,94]. This dysregulated uptake leads to accelerated foam cell formation, the core of the atherosclerotic plaque. Furthermore, systemic oxidative stress reduces LDLR expression in hepatocytes through epigenetic mechanisms, particularly via ROS induced methylation of its promoter [98,99,100].

Instead, some studies have underscored the critical role of cysteine residues in the functional regulation of transporter proteins. In the case of ABCA1, in vitro models have demonstrated that most of the cysteine residues require palmitoylation, involving the covalent attachment of palmitic acid, to ensure proper activity. Accordingly, mutations at residues Cys^3^, Cys^23^, Cys^1100^, and Cys^1111^ abolish palmitoylation at these sites and consequently result in a 40–60% reduction in the cholesterol efflux capacity of the transporter. In the case of ABCG1, studies in human cells have shown that mutations at Cys^26^, Cys^248^, Cys^298^, Cys^390^, Cys^397^, and Cys^402^ markedly impair its palmitoylation. Nevertheless, a significant decrease in cholesterol efflux has been experimentally confirmed only for mutations at Cys^298^ and Cys^402^ [101,102].

Advances in the understanding of modifications in these transporters offers a promising therapeutic approach for pharmacological targets that may contribute to the prevention and clinical management of cardiovascular diseases, as further discussed in the subsequent section.

## 5. Potential Treatments

As the activity of ABC transporters is essential for lipid homeostasis, their deregulation can cause multiple lipid-metabolism diseases, such as Tangier disease, sitosterolemia, atherosclerosis, metabolic syndrome, obesity, stroke, type 2 diabetes, and Alzheimer’s disease [29,103].

The conventional approaches to treating them include the use of drugs or traditional compounds that regulate the expression and stability of the ABCA1 and ABCG1 transporters [103], reducing lipid accumulation, avoiding atherosclerotic plaque formation, and increasing cholesterol efflux [104]. In Table 2, a comparison of the potential or available therapies is shown.

**Table 2:** Potential or available treatment for ABCA1 and ABCG1 transporter-related diseases.

Compound	Status	Nature	Target	Effect	Mechanism	Model System	Adverse Effects	Reference
Antagomir	Preclinic	Synthetic	ABCA1 & ABCG1	↑	↓ miR-23a-5p	ApoE^−/−^ mice	ND	[105]
Cilostazol	Clinic	Synthetic	ABCA1	↑	↑ LXR/ABCA1/SREBP-1	Human hepatoma cell line HepG2	ND	[106]
CoQ	Preclinic	Natural	ABCG1	↑	↑ Activator protein-1, ↑ miR-378	C57BL/6J mouse peritoneal macrophages, J774. A1, THP-1, HEK293 cells & ApoE^−/−^ mice	No found	[107]
Diosgenin	Preclinic	Natural	ABCA1	↑	↓ miR-19b,	THP-1 macrophages/MPM-derived foam cells & ApoE^−/−^ mice	ND	[108]
Dihydrogen	Clinic	Synthetic	ABCA1	↑	ND	Potential metabolic syndrome patients	No found	[109]
Hydrogen sulfide	Preclinic	Synthetic	ABCA1	↑	↑ Peroxisome proliferator-activated receptor α (PPARα) translocation	Human hepatoma cell line HepG2 & ApoE^−/−^ mice	ND	[110]
Paeonol	Preclinic	Natural	ABCA1	↑	↓ Calpain-related pathway, ↓ CD36 (platelet glycoprotein-4), ↑ Heme oxygenase-1	RAW264.7 macrophages & ApoE^−/−^ mice	ND	[104]
Qingre Sanjie Formula	Preclinic	Natural	ABCA1	↑	↑ LXRα/ABCG5/G8 pathway	ApoE^−/−^ mice	No found	[111]
Statins (Atorvastatin & pitavastatin)	Clinic	Synthetic	ABCA1	↓	↓ Protein kinase B phosphorylation, ↑ miR-33 levels	RAW264.7 cells & bone marrow-derived macrophages	Risk of hyperglycemia and new-onset diabetes	[112]
Triptolide	Preclinic	Natural	ABCA1	↑	May mediate expression through LPS/TLR4/GPS2 pathway	Male Sprague Dawley rats	ND	[113]

↑: upregulation, ↓: downregulation, ND: not determined.

Apart from ABCA1 and ABCG1, other components of the pathway can be targeted to increase the HDL levels; however, most of them cause several side effects, for example, LXR antagonist (LXR-623) administration is related to liver steatosis and neurological problems [114], and administration of external ApoA-1 with hepatotoxicity [115]. Nevertheless, the use of CETP inhibitors (such as anacetrapib and dalcetrapib) has been shown to increase HDL cholesterol levels without adverse effects, providing a safe option for patients with dyslipidemia [116,117]. The same occurs when Ezetimibe, an inhibitor of the Niemann-Pick-C1 protein (NPC1L1), is administered; the HDL cholesterol increases and LDL cholesterol and triglycerides levels decrease without adverse effects [118].

Moreover, the use of combination treatments without adverse effects has been reported. The work by Ma et al. (2018) indicates that when metformin is administered with T0901317 (a selective LXR inhibitor), their inhibitory effect on atherosclerosis is enhanced [119]. A similar effect occurs when niacin (vitamin B3) is applied together with statins [120].

In addition, a synthetic ApoE peptide called EpK, reduces the development of atherosclerosis by binding to HDL in ApoE-deficient mice [121]. When EpK is linked to methionine sulfoxide reductase A (MsrA), a hepatic protein with anti-atherosclerotic properties, its effect is enhanced due to the improvement of the redox status and inflammatory profile of dysfunctional HDL, promoting hepatic cholesterol uptake and excretion. EpK-MsrA also exhibited anti-inflammatory and modulatory effects on key HDL-associated proteins, including ApoA-I, paraoxonase 1 (PON1), and LCAT, leading to a further reduction in atherosclerotic burden [35].

On the other hand, an emerging strategy is the use of miRNAs in the regulation of ABCA1 and ABCG1 transporters expression [122]. miRNAs are small non-coding RNAs that control gene expression due to the binding of 3′UTR regions of mRNAs [123]. Many miRNAs have been reported to modulate lipoprotein metabolism. In Table 3, a compilation of these compounds and their effect on transporter gene expression is shown. For further information, we refer the reader to Rozhkova et al. (2021) [25] and Goedeke et al. (2014) [124].

Furthermore, some natural antioxidant compounds have been used as a promising treatment for atherosclerosis: *Auricularia heimuer*, better known as heimuer, with its anti-inflammatory, hypolipidemic, and antioxidant effects, demonstrates regulating lipid levels, gut microbiota, body weight, and alleviating aortic lesions in atherosclerosis induced in a high-fat diet ApoE^−/−^ mice [125]. Curcumin, normally consumed for its anti-inflammatory properties, also modulates gut microbiota with anti-obesity and anti-hyperlipidemic effects that contribute to the prevention atherosclerosis progression [126]. Resveratrol attenuates the progression of atherosclerosis by inhibiting the oxidative modification of LDL cholesterol and demonstrates multifaceted anti-atherosclerotic effects [127]. Oleuropein, extracted from olive leaves, significantly attenuated the atherosclerosis progression and enhanced plaque stability. It is demonstrated that this compound suppresses ferroptosis by upregulating GPX4/xCT (Phospholipid hydroperoxide glutathione peroxidase 4/Cystine/glutamate transporter) level and enhances Nrf2 activation and nuclear translocation [128]. Finally, brown marine algae-derived polysaccharides, such as fucoidan, alginate, laminarin, carrageenan, and chitosan, exert anti-inflammatory, antioxidant, lipid-regulating, antithrombotic, and endothelial-protective effects, relevant to the pathogenesis of atherosclerotic cardiovascular disease, but their oral bioavailability and structural diversity are challenges to overcome for using them for preventing and treatment of atherosclerotic cardiovascular disease [129].

One of the main challenges of current therapies, regardless of their chemical nature, lies in the adverse side effects on the body. However, the development of nanomaterials offers a promising option to overcome these limitations [130]. For example, a nanofibrous hydrogel encapsulating D-Nap-GFFY-T0901317, an LXR agonist, has been shown to inhibit atherosclerosis without inducing hepatic lipogenesis, representing a novel and potentially effective strategy for the treatment of atherosclerosis [131].

## 6. Limitations and Perspectives

ABCA1 and ABCG1 transporters are potential pharmacological targets for the prevention and treatment of atherosclerosis. However, safe, effective clinical therapies without serious side effects are not yet available. Therefore, it is necessary to develop molecules that specifically activate ABCA1 and ABCG1 through genetic regulation.

To date, attempts to induce ABCA1 and ABCG1 expression by modulating LXR have been unsuccessful due to adverse effects. For example, administration of LXR-623 (an LXR antagonist) that increases mature HDL levels may cause hepatic steatosis and neurological problems [114]. Consequently, current research focuses on engineering drugs that can target ABCA1 and ABCG1 expression through transcriptional regulation, such as the PPAR-α-based therapies [110]. One potential strategy is to combine both compounds to enhance their effect on atherosclerosis. LXR agonists induce ABCA1 and ABCG1 expression, while PPAR stimulation suppresses triacylglycerol levels by increasing β-oxidation and LIPC enzyme activity, thereby avoiding potential serious side effects [77,78].

Another approach is the development of compounds that specifically increase the activity of ABCA1 and ABCG1 or prevent their protein degradation. For example, the administration of a recombinant HDL-like particle (designated ETC-216), containing ApoA-I, resulted in a 4.2% decrease from baseline in coronary atheroma volume measured by intravascular ultrasound [132].

On the other hand, numerous reports describe several natural products present in foods, as well as synthetic small molecules, that could activate the expression of ABCA1 and ABCG1. Still, most of them are in the preclinical stage (see Table 2), so greater effort is needed to implement them in the clinic.

Furthermore, the origin of ABCA1 and ABCG1 dysfunction lies in the cysteine mutation experiments [101,102]; these residues may serve as susceptible targets for oxidation, given their high reactivity under prooxidant conditions. This could account for the reduced activity of ABCA1 and ABCG1, clarifying the pathophysiology of atherosclerosis and potential therapeutic targets.

The next critical step in understanding the pathophysiology of atherosclerosis involves characterizing the precise structural and functional consequences of oxidative stress on ABCA1 and ABCG1. Given the complexity and range of potential modifications, bioinformatic tools can generate high-resolution structural models, predict the dynamic effects of cysteine oxidation at the atomic level, and identify new susceptible residues. Additionally, the genetic and epigenetic regulation of ABCA1 and ABCG1 expressions represent a promising approach to restore HDL function, potentially with a lower risk of systemic adverse effects than direct pharmacological interventions. Finally, integrating these computational insights with in vitro functional assays could facilitate the development of compounds that enhance antioxidant defenses or stabilize transporter activity, which represents a future direction for reducing atherosclerotic damage and the burden of cardiovascular disease.

## 7. Conclusions

The present work aimed to provide a concise overview of HDL metabolism, emphasizing the key roles of the ABCA1 and ABCG1 transporters and their correct function, which is currently of greater relevance due to increasing scientific evidence supporting the notion that oxidative stress promotes their dysfunction, thus contributing to the development of cardiovascular diseases such as atherosclerosis. However, the precise molecular mechanisms underlying this impairment remain unclear, and based on the data available to date, ABCA1 and ABCG1 could be good candidates as pharmacological targets to induce proper RCT function. Safer and more effective clinical treatments are needed to reduce side effects in patients with atherosclerosis or related conditions characterized by low circulating HDL levels.

## Figures and Tables

**Table 3:** Effect of miRNAs on the expression levels of ABCA1 and ABCG1 transporters.

Transporter	ABCA1		ABCG1	
Effect	Upregulation	Downregulation	Upregulation	Downregulation
miRNA	miR-28	miR-10b, miR-17, miR-19b, miR-20, miR-23a-5p, miR-26, miR-27, miR-30e, miR-33, miR-34a, miR92a, miR-93, miR-101, miR-106b, miR-128, miR-130, miR-143, miR-144, miR-145, miR-183, miR-301b, miR302a, miR-361-5p, miR-613, miR-758 * miR-1, * miR-128, * miR-155, * miR-212, * miR-223, * +miR-206, * miR-486	ND	miR-10b, miR-23a-5p, miR-33, miR-34a, miR378

* The effect of these miRNAs is not directly on the transporter, but on a related protein that regulates its function.

## Data Availability

No new data were created or analyzed in this study. Data sharing is not applicable to this article.

## Abbreviations

The following abbreviations are used in this manuscript:

HDLs	High-density lipoproteins
ABC	ATP-binding cassette
LDLs	Low-density lipoproteins
CMs	Chylomicrons
VLDL	Very-low-density lipoprotein
RCT	Reverse cholesterol transport
ROS	Reactive oxygen species
NBDs	Nucleotide-binding domains
TMDs	Transmembrane domains
ECDs	Extracellular domains
LXR	Liver X receptor
RXR	Retinoid X receptor
PPAR α and γ	Peroxisome proliferator-activated receptors alpha and gamma
SREBF1	Sterol regulatory element binding factors 1
SREBF2	Sterol regulatory element binding factors 2
AD	Alzheimer’s disease
miRNAs/miR	MicroRNAs
LCAT	Lecithin cholesterol acyltransferase
PC	Phosphatidylcholine
UC	Unesterified cholesterol
PLs	Phospholipids
SR-B1	Scavenger receptor class B type 1
CETP	Cholesteryl ester transfer protein
PLTP	Phospholipid transfer protein
LIPC	Hepatic lipase
LIPG	Endothelial lipase
SREBP-2	Sterol regulatory element-binding protein 2
HMG-CoA	β-hydroxy β-methylglutaryl-CoA
HMGR	HMG-CoA reductase
HMGS	HMG-CoA synthase
Sqs	Squalene synthase
Fpps	Farnesyl diphosphate synthase
LDLR	Low-density lipoprotein receptor
INSIG1	Insulin-induced gene 1 protein S
StarD4	StAR-related lipid transfer protein 4
Nrf2	Nuclear factor erythroid 2
SR-A1	Scavenger Receptor Class A type 1
CD36	Platelet glycoprotein 4
NPC1L1	Niemann-Pick-C1 protein
MsrA	Methionine sulfoxide reductase A
PON1	Paraoxonase 1
GPX4/xCT	Phospholipid hydroperoxide glutathione peroxidase 4/Cystine/glutamate transporter
